# Finite Element Combined Design and Material Optimization Addressing the Wear in Removable Implant Prosthodontics

**DOI:** 10.3390/jfb15110344

**Published:** 2024-11-14

**Authors:** Pejman Shayanfard, Xingchen Tan, Matthias Karl, Frank Wendler

**Affiliations:** 1 Department of Materials Science, Institute of Materials Simulation, Friedrich-Alexander University Erlangen-Nürnberg, 90762 Fürth, Germany; pejman.shayanfard@fau.de (P.S.);; 2 Department of Prosthodontics, Saarland University, 66421 Homburg, Germany; matthias.karl@uks.eu

**Keywords:** implant-supported removable prosthesis, wear in female attachment part, titanium, shape memory alloy, design optimization, material optimization, finite element method

## Abstract

Wear at the male–female interface of retentive elements in implant-supported removable prostheses is the most frequent complication in such applications. The lack of an ideal/optimal insertion path, as well as the fabrication inaccuracies, are the primary contributors to this issue. A male attachment with a common ball anchor enhanced by lateral flexibility was investigated as a solution, compared to the widely used rigid ball anchor design. A parametric finite element analysis was performed to compare the wear-inducing maximum strain at the female polymer counterpart by various attachment designs made from titanium and Nitinol. The evolution of mechanical strains causing wear in the female part, as well as the contribution of stresses and martensitic transformation in the implant’s flexible shaft, were evaluated under several insertion misfit scenarios. Results indicate that introducing a long flexible shaft in the titanium implant reduced maximum strains in the female attachment part by up to 61% as compared to the solid ball anchor. Further improvement was observed by using the shape memory alloy Nitinol as shaft material, leading to a minor reduction in stress and strain at the contact surface but allowing for a shorter abutment. Finally, the optimized Nitinol implant design with a short, necked flexible shaft promoting martensitic transformation at low plateau stress resulted in an approximate 90% reduction in maximum strains at the inner surface of the female part during manual insertion, which indicates a significantly reduced wear phenomenon at the contact.

## 1. Introduction

In recent years, shape memory alloys (SMAs), particularly nickel–titanium alloys (Nitinol), have found extensive applications in medical technology [1,2]. Nitinol first established its presence in the medical market through its use in stents—vasodilating meshes that support arteries and veins [3,4,5,6]. Additionally, SMAs are frequently utilized in the dental industry [7], notably in brace wires designed to apply constant tension over large deformation paths of the teeth and in root canal files, which leverage the special properties of Nitinol to navigate curves without breaking [8,9]. Nitinol’s biocompatibility [10,11] and unique characteristics have significantly enhanced the quality of life for millions of patients. In vascular surgery, superelastic Nitinol stents stabilize coronary vessels minimally invasively, while superelastic brace wires maintain a constant tensile force between teeth.

Given the remarkable properties and successful applications of shape memory alloys (SMAs) in various medical fields, their potential to address persistent challenges in dental prosthodontics is promising. The unique capabilities of SMAs, particularly their superelasticity and biocompatibility, offer innovative solutions to improve the performance and longevity of dental attachment systems. Integrating SMAs into the design of removable implant-supported prostheses makes it possible to mitigate common issues such as wear and misfit at the male–female interface, which are critical factors influencing prosthesis durability and patient satisfaction.

**Figure 1:**
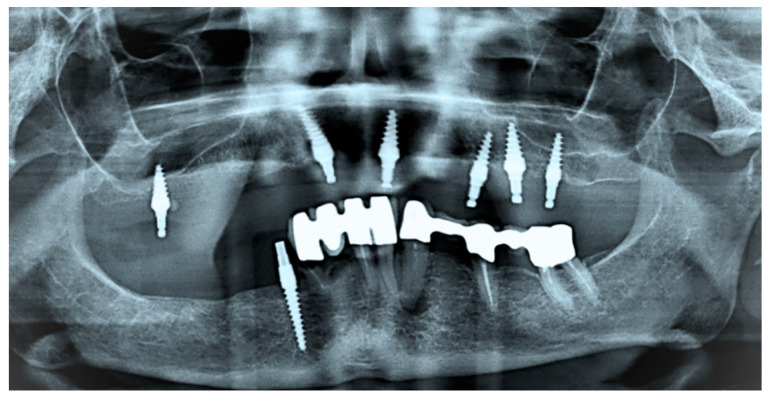
Panoramic radiograph of a geriatric patient presenting multiple single-piece implants in the maxilla with balls for prosthesis retention (implant placement alio loco several years ago). Please note that a common path of insertion has not been established for the maxillary implants. The mandibular dentition is failing, but the patient still wanted to postpone treatment.

**Figure 2:**
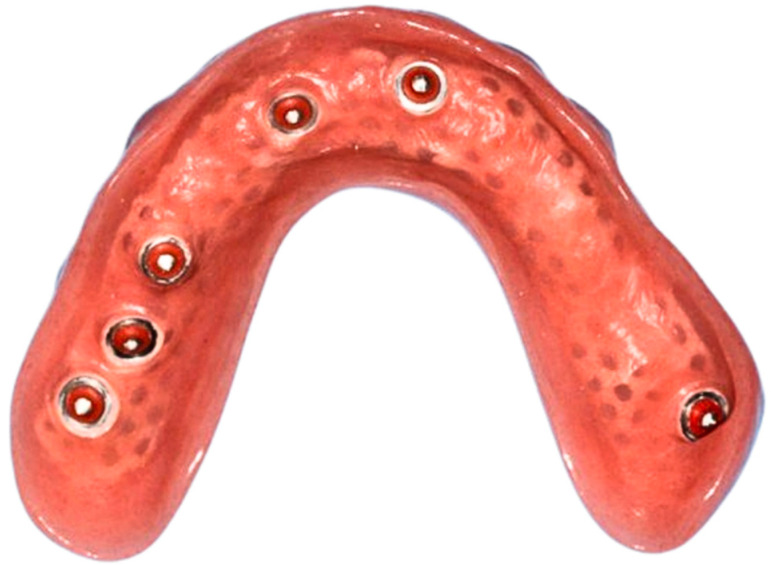
Maxillary removable prosthesis not covering the palate as per the patient’s request. New O-rings have been placed inside corresponding metal housings to fit onto the implants. Two O-rings were intentionally removed as the patient was unable to remove the restoration when all six balls were engaged.

**Figure 3:**
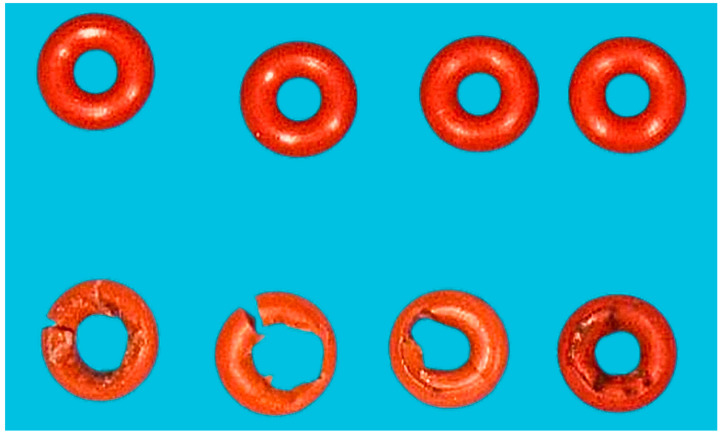
Eight months after prosthesis delivery, the patient complained about loss of retention of her restoration, presenting with the O-rings shown in the bottom line (top line: new O-rings for comparison). Unilateral deterioration of the O-rings following compression of the material during repeated insertion and removal of the prosthesis is obvious.

Removable implant-supported prostheses have transformed modern dentistry by providing patients with comfortable and functional alternatives for tooth replacement, especially in edentulous mandibles [12,13]. However, the persistent issue of component wear at the male–female interface of attachment systems [12,14] used for retaining implant-supported removable prostheses remains a significant concern in clinical practice, causing considerable maintenance costs [15,16]. With individually fabricated options such as telescopic crowns [17,18] and bars [19,20] often being considered cost-prohibitive, prefabricated single-standing components such as ball anchors (Figure 1 and Figure 2) and locators have been adopted more widely. Wear phenomena (Figure 3) not only jeopardize the longevity and effectiveness of the prosthetic device but also lead to patient discomfort and dissatisfaction, constituting the major complication reported in removable implant prosthodontics [21].

A notable example is the higher wear observed in titanium-to-titanium attachment systems, which showed substantial abrasion and accumulation of metallic particles due to friction [22]. In contrast, attachments with dissimilar materials, such as the Locator R-Tx with a titanium-to-nylon interface, exhibited pronounced wear in the softer nylon matrix, reducing wear on the metal patrix and offering potentially longer-lasting retention [23]. Major contributing factors to wear include impression inaccuracies [24,25] and non-parallelism [26,27] of supporting implants (Figure 1). Industry efforts to address these challenges have involved the introduction of angulated abutments [28], variations in materials for the female part [29], and improving the surface topography of male attachment parts, e.g., through the application of diamond-like carbon coatings to the abutments [30].

Studies indicate that resilient attachments show significant wear on plastic components after cyclic loading, resulting in a marked drop in retention force, sometimes up to 88% [31]. Clinical observations of O-ring attachments further highlight wear-induced retention loss, typically requiring replacement every 6–9 months, especially under stress conditions such as misalignment or environmental factors [32]. Additionally, ball attachments with O-rings made from materials like nitrile rubber show increased wear and retention loss in non-parallel setups, where lateral forces and high angulation exacerbate degradation [33].

Material selection and environmental considerations play crucial roles in wear dynamics; studies on rubber–metal interactions reveal that inert atmospheres accelerate metal wear significantly, indicating that oxygen presence can stabilize rubber-induced metal wear by limiting radical formation [34]. For spherical attachments, implant angulation directly affects retention, with higher angulation correlating with greater wear due to increased friction and uneven force distribution [35].

Research comparing prefabricated spherical and cylindrical attachments underscores the design implications for wear reduction, advocating simpler attachment designs with frictional stability over their spring-loaded counterparts, which experience rapid retention loss [36]. Clinical trials on mandibular overdentures suggest that stress-relieving bar attachments help reduce wear on O-rings, yet still require periodic maintenance due to retention loss from gradual O-ring deformation [37]. Lastly, in vitro evaluations comparing O-ring and Locator attachments affirm that, while Locator attachments provide higher and more stable retention, they also show greater resilience to wear under axial forces compared to O-rings, which wear faster under lateral dislodging stresses [38]. This evidence underscores the significance of material choice, alignment precision, and attachment design in mitigating wear-related complications in implant-supported removable prostheses.

Additional studies further emphasize the complexities of wear in implant-supported systems. For instance, biomechanical evaluations of various attachment types and implant positions in mandibular single-implant prostheses (MSIP) reveal that no single attachment system minimizes wear optimally, as strain is influenced by both attachment type and implant positioning [39]. Furthermore, innovative NiTi-based flexible attachments show promise by reducing strain on implants and addressing positional discrepancies, thereby potentially enhancing both longevity and patient satisfaction [40]. The adaptability and durability of these NiTi systems [41] underscore their capability to significantly reduce wear-associated concerns in implant-supported overdentures.

To build on the findings discussed, it is clear that addressing wear phenomena in attachment systems is critical for improving prosthesis longevity, patient comfort, and satisfaction. Given the persistent challenges of retention loss due to wear and the limitations of current attachment materials and designs under non-parallel conditions, a novel approach could involve utilizing attachment systems with flexible male components capable of compensating for positional errors and non-parallelism [42,43]. Previous work has demonstrated that such attachment systems utilizing shape memory alloys [44] optimize the loading conditions of the supporting implants [42] and eliminate the dependence of prosthesis retention on misfit phenomena. To predict the potential benefits of such an attachment system in terms of prosthesis performance and longevity, it is the goal of this parametric study using finite element method (FEM) simulations [45] to analyze the effects of attachment design, material properties, and misfit between supporting implants and prosthesis [46] on the stress–strain distribution within the postulated attachment system.

## 2. Materials and Methods

### 2.1. FEM Model

Based on an existing ball anchor attachment (Clix, Hader Solutions, Dublin, Ireland), a one-piece implant including the male attachment part was modeled in four different designs (see Table 1).

**Figure 4:**
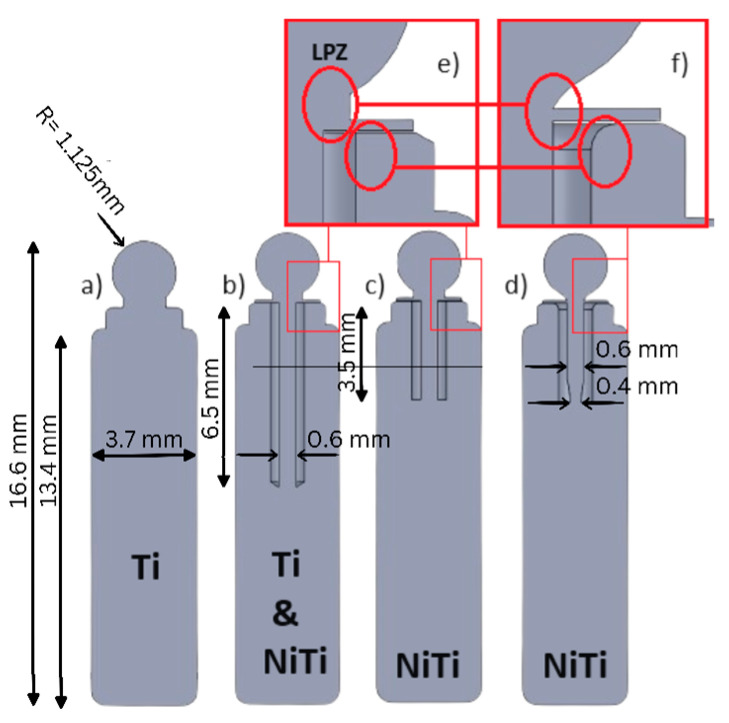
(**a**) Reference: solid Ti implant; (**b**) Ti and Nitinol implants, where a flexible long shaft is embedded; (**c**) short Nitinol implant, where the embedded long shaft is 3 mm shorter; (**d**) short-necked Nitinol implant; (**e**) representation of a design fault zone denoted as LPZ within the manuscript; and (**f**) representation of the optimized design in LPZ for the short-necked Nitinol implant.

**Figure 5:**
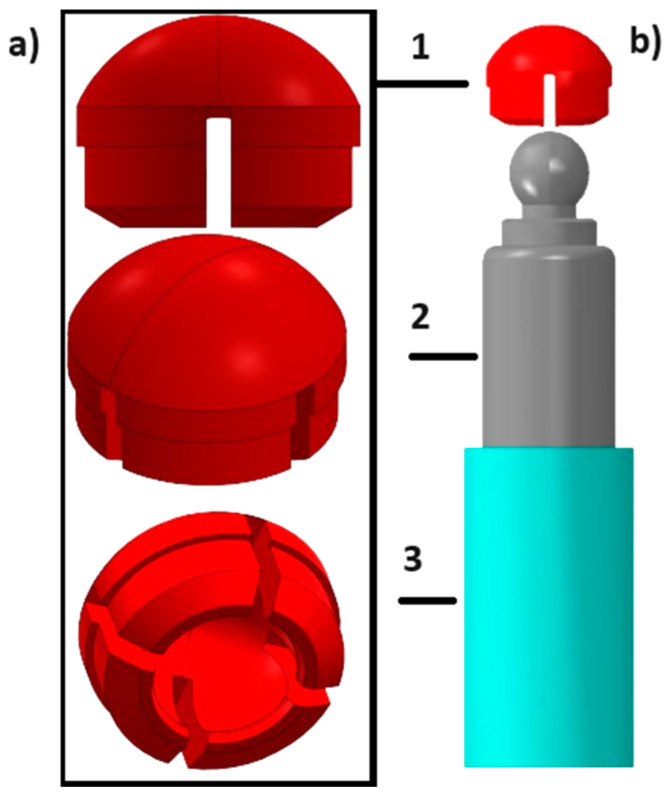
Three-dimensional model of a simplistic one-piece attachment with a ball anchor on top for retaining a removable prosthesis; (**a**) a 3D model of the female part fitting the ball anchor in different views; and (**b**) representation of the entire assembled FEM model including the surrounding Ti abutment: 1—female part; 2—male attachment part; and 3—Ti implant.

As a reference, a solid attachment without any flexibility was modeled, representing the current state of the art (Figure 4a), and three configurations where the ball was retained by a centrally positioned, rod-shaped shaft allowing for lateral movements of the ball anchor (Figure 4b–d). A secondary (female) component to be mounted in the removable prosthesis, matching the ball (Figure 5a), was also modeled. The titanium implant placed in the bone is added (part number 3 in Figure 5b) in a surface-to-surface contact method to follow the effects of the material and design change in the implant on the induced reaction forces on the abutment.

**Table 1:** Overview of abutment designs, misfit configurations, and loading situations used in this parametric FEM study.

Abutment Type	Misfit Type (Figure 6)	Loading Situation
Solid Ti (Figure 4a)	Perfect fit, horizontal misfit, angulation misfit	Maximum, Final
Long Ti (Figure 4b)	Angulation misfit	Maximum, Final
Long Nitinol (Figure 4b)	Angulation misfit	Maximum, Final
Short Nitinol (Figure 4c)	Angulation misfit	Maximum, Final
Necked Nitinol (Figure 4d)	Angulation misfit	Maximum, Final

**Figure 6:**
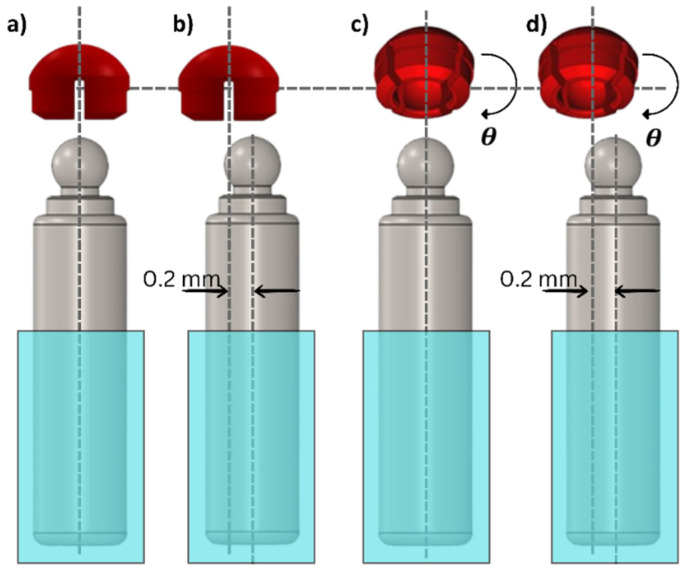
Misfit types considered in this study: (**a**) perfect fit; (**b**) horizontal misfit between male and female attachment parts of 0.2 mm; (**c**) angulation misfit with the female part rotated (θ= 3 degrees); and (**d**) horizontal and angulation misfit.

Given the inevitable but unknown positional discrepancies between implant and prosthesis resulting from the insertion process, four misfit scenarios [46] were simulated (Table 1 and Figure 6, see the movies in the supporting materials to this article). In the perfect fit situation (Figure 6a), the longitudinal axis of the female component and the one-piece implant are aligned. In the horizontal misfit situation, the abutment was shifted horizontally by 0.2 mm (Figure 6b), while for angulation misfit the abutment was rotated 3 degrees (Figure 6c). In addition, a combination of horizontal and angulation misfits was introduced to better resemble clinical reality; this was only used at the final stage to cover the analysis of the material and design optimizations on the reaction forces induced on the surrounding abutment (Figure 6d). For the design and material optimization, only the angulation misfit was used. This is in line with previous research works, where an angular mismatch was identified as the most critical contribution to wear, as compared to vertical or horizontal mismatch [33,46].

The female part is meshed using C3D4H 4-node linear tetrahedron elements, employing a hybrid formulation and linear geometric order. It consists of approximately 60,000 elements and 12,500 nodes. The implant utilizes two types of elements: C3D10, a 10-node quadratic tetrahedron element, is used for parts in contact with the female part and the flexible shaft experiencing major deformations; C3D4, a linear 4-node tetrahedron element, is used in the low-stress regions of the implant. The number of nodes and elements in the final design varies slightly, approximately 25,000 nodes and 50,000 elements, depending on the specific design. All simulations in this study employ curve-following discretization refinement to ensure result accuracy.

### 2.2. Materials

The abutment types “Solid Ti” and “Long Ti” were modeled using the material properties of titanium (Young’s modulus 116 GPa; Poisson’s ratio 0.34), while the design variants of the flexible abutment types were realized in Nitinol. The superelastic behavior of the Nitinol implants in this study is governed by key parameters such as the start and end of transformation in loading ($\sigma_{s}^{Cr,\;load},\sigma_{f}^{Cr,\;load}$), the start and end of transformation in unloading ($\sigma_{s}^{Cr,\;unload},\sigma_{f}^{Cr,unload}$), elastic moduli for austenite (E_A_) and martensite (E_M_), Poisson’s ratios (*ν*_*A*_ and *ν*_*M*_), and transformation strain (*ϵ*^*T*^). These values were adapted from tensile test data provided by the manufacturer of the prototype NiTi attachment in our previous study [43]. The tension–compression asymmetry in the elastic moduli and stress plateaus were assumed to match typical values reported in the literature [45,46]; this asymmetry is essential for understanding the deformation mechanism under bending loads. Table 2 presents the material properties of the medical-grade Nitinol, experimentally obtained from our prior study [43]. The material selected for the female part was polyoxymethylene (POM; Table 3), for which an elastic-plastic material model was implemented, obtained by parameter fitting to the stress-strain data plotted at 40 °C in Figure 5 of the reference [47] (see Table 4).

### 2.3. Boundary Conditions

The one-piece abutment was rigidly fixed along the lateral axis, simulating the constraints imposed by the surrounding bone on both the implant’s and the abutment’s movement. Similarly, the female attachment part was immobilized to mimic the constraints imposed by a removable prosthesis. A general contact with a friction coefficient of 0.1 was established to mimic the contact between the abutment and the implant and between the implant and the female part.

To simulate the insertion and removal of the prosthesis by the patient, the bottom surface of the abutment is displacement-controlled along the axial direction.

Two different loading situations (Table 1) were considered in this study to resemble the insertion process of the prosthesis. The ‘Maximum’ loading situation was defined as the moment when the strain at the edges of the female part reached its peak during the insertion process, coinciding with the maximum deformation of the female plastic inserts. The ‘Final’ loading situation was defined as the conclusion of the insertion process, i.e., when the ball anchor was fully inserted into the female part. Additionally, the displacement was further increased by 0.1 mm to simulate an extra force exerted by the patient, e.g., representing the chewing force.

## 3. Results and Discussion

### 3.1. The Effects of the Design and Material Optimization on the Mechanical Fields in the Female Part

**Figure 7:**
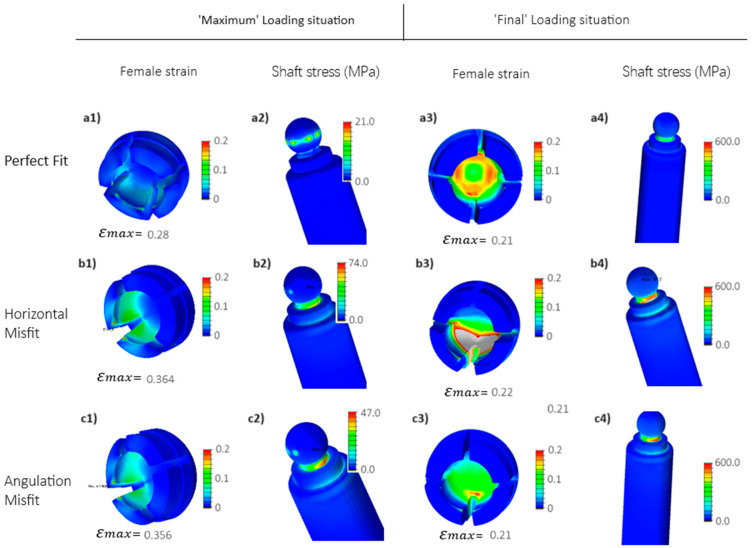
FEM results for the reference solid Ti implant design. Stresses are depicted as equivalent to von Mises stress, while strains represent maximum principal strain; (**a1**–**a4**) perfect fit (see Figure 6a), where the longitudinal axis of the female component aligns with that of the inserted implant; (**b1**–**b4**) horizontal misfit: the abutment shifts horizontally for 0.2 mm (see Figure 6b); and (**c1**–**c4**) angulation misfit: the abutment rotates for 3 degrees (see Figure 6c).

**Figure 8:**
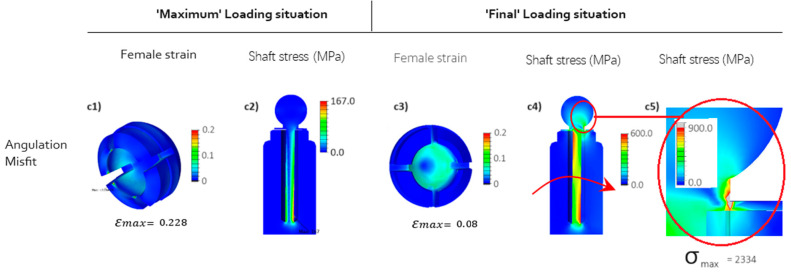
FEM results for the angulation misfit (see Figure 6c) of the long Ti implant (see Figure 4b). Stresses are depicted as equivalent to von Mises stress, while strains represent the maximum principal strain. (**c1**–**c4**) angulation misfit: the abutment rotates for 3 degrees (see Figure 6c), (**c5**) Detail of the flexible ball head (stress).

**Figure 9:**
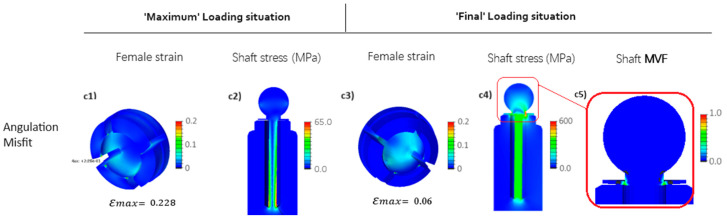
FEM results for the long Nitinol implant, featuring an embedded flexible Nitinol long shaft (see Figure 4b) for the angulation misfit (see Figure 6c). Stresses are depicted as equivalent to von Mises stress, while strains represent the maximum principal strain. In (c5), the martensite volume fraction is mapped. (**c1**–**c4**) angulation misfit: the abutment rotates for 3 degrees (see Figure 6c), (**c5**) detail of the flexible ball head (martensite volume fraction MVF).

**Figure 10:**
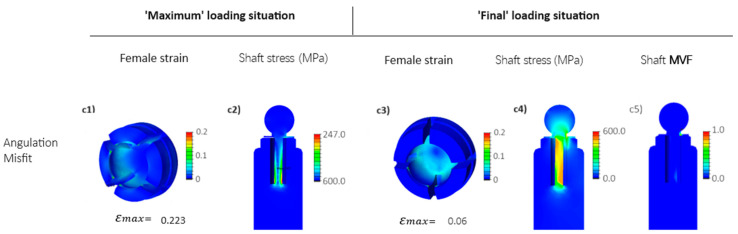
FEM results for the short Nitinol implant, featuring an embedded flexible Nitinol short shaft (see Figure 4c) for the angulation misfit (see Figure 6c). Stresses are depicted as equivalent to von Mises stress, while strains represent the maximum principal strain. (**c1**–**c4**) angulation misfit: the abutment rotates for 3 degrees (see Figure 6c), (**c5**) Martensite volume fraction MVF.

**Figure 11:**
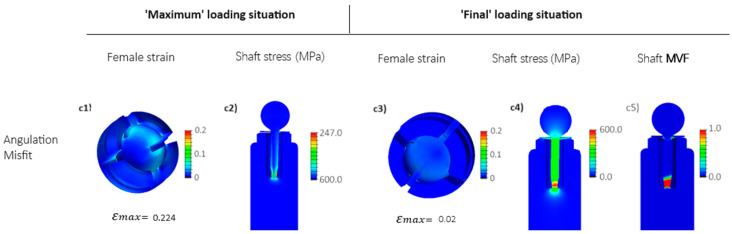
FEM results for the short-necked Nitinol implant, featuring a short-necked embedded Nitinol shaft (see Figure 4d) in angulation misfit (see Figure 6c). Stresses are depicted as equivalent to von Mises stress, while strains represent the maximum principal strain. (**c1**–**c4**) angulation misfit: the abutment rotates for 3 degrees (see Figure 6c), (**c5**) Martensite volume fraction MVF.

Figure 7, Figure 8, Figure 9, Figure 10 and Figure 11 comprehensively represent the results, showcasing the spatial distributions of stress, strain, and martensite volume fraction (where applicable) across all parameters.

#### 3.1.1. Solid Ti Implant

The results indicate that, during the ‘Maximum’ loading situation, the edges of the female part experience the highest strain level, approximately reaching 0.36% (Figure 7) when the implant is horizontally misfitted or has an angulation misfit (refer to Figure 7(b1,c1)), compared to 28% strain in the perfect fit case study shown in Figure 7(a1). This underscores the impact of non-ideal alignment between the axes of the implant and the female part. Since the angulation misfit represented a worst-case scenario as compared to horizontal and vertical mismatches [46], it was exclusively used for subsequent design and material optimization iterations.

#### 3.1.2. Long Ti Implant

Figure 8 illustrates the results for the upgraded Ti implant design, where the implant design is modified to facilitate deformation by creating a flexible central shaft (see Figure 4b). At the ‘Maximum’ loading situation (depicted in Figure 8(c1)), it is evident that the flexible design of the implant’s central shaft notably diminishes the maximum induced strain at the edges of the female part during insertion (‘Maximum’ loading situation), reducing it from approximately 36% (Figure 7(c1)) to about 22.8%, which is a 40% reduction. However, due to increased deformation freedom, the shaft experiences a stress of 167 MPa (as shown in Figure 8(c2)), significantly higher as compared to the stress depicted in Figure 7(c2), attributable to the linear Hook’s stress–strain relation.

Furthermore, in the ‘Final’ loading situation, which realizes an additional imprint force after ball anchor insertion, the results demonstrate that this initial design improvement leads to a significant reduction in the maximum induced strain at the inner surface of the female part. Specifically, there is a notable decrease from approximately 21% maximum strain in Figure 7(c3) to around 8% in Figure 8(c3), representing roughly a 61% reduction. However, the simulation results for the shaft’s stress at the ‘Final’ loading situation (as depicted in Figure 8(c4,c5)) reveal a design flaw in the improved flexible configuration. Notably, the stresses at the sealing disk of the implant exceed 2 GPa, indicating a potential risk of tearing and preventing further flexibility of the shaft. This pivotal design area is henceforth denoted as the Locking Point Zone (LPZ) throughout the remainder of this manuscript, as it will receive further attention later on during design and material optimization progress.

#### 3.1.3. Long Nitinol Implant

Here, superelastic Nitinol is used as the material of the long shaft design (see Figure 4b). Critical stress at the LPZ is partially addressed by employing Nitinol material, which reduces the maximum stress level at the LPZ to around 600 MPa (compare Figure 8(c4) and Figure 9(c4)), as the phase transformation occurring in Nitinol allows for a high degree of deformation at the plateau stress level. This is evident when observing the martensite volume fraction evolved at the LPZ depicted in Figure 9(c5). Nevertheless, the transition from Ti into Nitinol material for the long embedded shaft does not result in any significant change in the maximum strain experienced at the edges of the female part during the ‘Maximum’ loading situation (compare Figure 8(c1) and Figure 9(c1)). This is because the maximum stress level reached at the shaft during this loading situation remains well below the martensitic phase transformation plateau stress (refer to Figure 9(c2)), so that the superelasticity of Nitinol does not occur during the deformation process at the ‘Maximum’ loading situation.

In other words, the deformation of the shaft is not big enough for Nitinol to transform into martensite at the ‘Maximum’ loading situation. However, the maximum strain reduction of the female part at the ‘Final’ loading situation, from approximately 8% to about 6%, equating to a 25% reduction (compare Figure 8(c3) and Figure 9(c3)), is attributed to a slight martensitic phase transformation at the LPZ of the implant (see Figure 9(c5)), facilitating increased flexibility of the shaft. The results indicate only a marginal improvement when changing from elastic Ti to NiTi as the material of the long flexible shaft. The advantage of the superelastic NiTi comes into play in the following design, which requires less vertical space in the implant.

#### 3.1.4. Short Nitinol Implant

In an attempt to manipulate the stress levels in the Nitinol shaft, the flexible shaft was shortened (see Figure 4c), leading to increased stress levels in the shaft in both loading situations, ‘Maximum’ and ‘Final’. Although the induced stresses in the shaft were successfully heightened, they remained within the elastic regime, i.e., below the martensitic transformation plateau stress at either loading situation (see Figure 10(c2,c4)). This means that the triggered stresses in the shaft were not high enough for Nitinol to transform into martensite. As a result, there were no significant changes in strain observed in the female part (see Figure 9(c1) vs. Figure 10(c1) and Figure 9(c3) vs. Figure 10(c3)).

#### 3.1.5. Short-Necked Nitinol Implant

To enhance the flexibility of the superelastic Nitinol shaft, a weak point was deliberately incorporated through cross-section reduction. This weak point was strategically introduced into the stress concentration pivotal zone of the shaft (see Figure 4d), where the bending–compression-induced strains are shown to be maximal, as demonstrated in the previous simulation (refer to the shaft’s stress distributions in Figure 8, Figure 9 and Figure 10). This technique, commonly referred to as necking in mechanics, allows the shaft to endure elevated local stress levels under an equivalent deformation history, i.e., at an equivalent nominal axial reaction force. Consequently, it promotes a greater degree of martensitic transformation evolution in the deformation process of the shaft. In addition, the deformation blocking in the LPZ was addressed by introducing local fillets (see Figure 4f).

While Figure 11(c2) shows slightly higher stress levels developed in the shaft at the ‘Maximum’ loading situation, it remains within the elastic regime below the martensitic transformation stress plateau, thus not affecting the maximum strain experienced at the edges of the female part during this loading situation. However, this design modification demonstrates a notable contribution of the martensitic transformation in the shaft’s deformation at the necked region at the ‘Final’ loading situation (refer to Figure 11(c5)), leading to significant strain relaxation in the female part to approximately 2% (Figure 11(c3)), compared to around 8% in Figure 10(c3). This represents a ~75% strain reduction compared to the long Nitinol implant and short Nitinol implant, and a ~90% reduction compared to the solid Ti implant, where the maximum strains experienced at the female part in the ‘Final’ loading situation could reach up to 21% (compare Figure 7(c3) to Figure 11(c3)).

### 3.2. The Effects of the Design and Material Optimization on the Abutment Reaction Stresses

**Figure 12:**
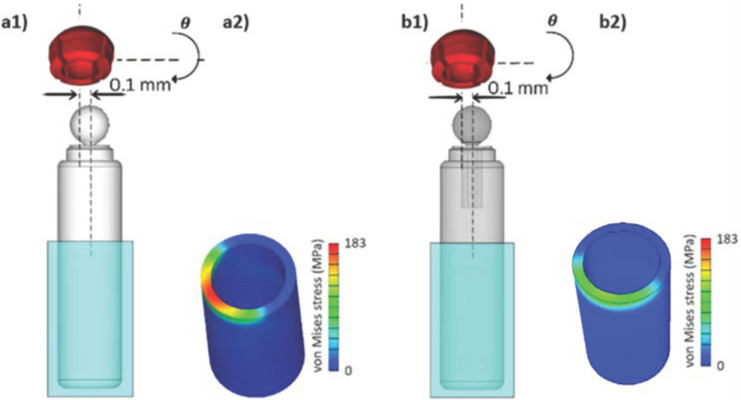
The effect of the ultimate design and material optimization on the reaction stresses on the surrounding Ti abutment. (**a1**,**b1**) The solid Ti implant and the flexible-necked Nitinol implant, respectively. In both trials, the implant is rotated (θ=3 degrees) and shifted horizontally by 0.1 mm in relation to the female part. (**a2**,**b2**) The resultant reaction stresses on the surrounding Ti abutment for the reference solid Ti design and the final optimized Nitinol implant design, respectively.

For a comprehensive investigation of the effect of the design–material optimization journey, the resultant reaction force on the surrounding Ti abutments is compared between the solid Ti implant and the short-necked Nitinol implant. To this end, a similar misfit between the female part and the implant, combining horizontal and angulation misfits [46], is considered during the insertion (see Figure 6d, equally represented in Figure 12(a1,b1)).

The findings demonstrate a reaction stress reduction from 183 MPa in the reference solid implant design (see Figure 12(a2)) to ~110 MPa in the final optimized implant design (see Figure 12(b2)), marking a significant 39% performance improvement.

In summary, based on the assumption that wear phenomena at the male–female interface of current attachment systems used for retaining removable implant-supported prostheses are due to misalignment of the supporting implants and fabrication inaccuracies [24,25,26,27], an attachment system incorporating a flexible male component was proposed [42,43]. Using a systematic combined design–material optimization approach, this finite element analysis [45] aimed to optimize the male attachment part concerning the deformations occurring in the female retentive components under different misfits [46] and loading situations. Compared to an existing solid ball anchor, incorporating an embedded flexible long shaft led to a significant (~40%) reduction in maximum strain on the edges of the female part during the insertion process. Additionally, a notable (~61%) reduction in strain was observed upon completion of the insertion process. Switching the shaft material from titanium to superelastic Nitinol only led to a minor reduction in the female part’s critical strain at the maximum loading situation, but further reduced the maximum strain at the final loading situation by about 25%. Finally, shortening the flexible Nitinol shaft did not yield substantial changes in strain levels, while a necked central shaft allowed further improvements in strain reduction during the final insertion process, facilitating a ~90% strain reduction compared to the solid ball anchor. Future research should focus on assessing the dynamic effects of chewing forces on wear, as well as the long-term performance of Nitinol components under clinical conditions, using dedicated experimental approaches and FEM packages like FEMFAT. Indeed, from a clinical perspective, it seems difficult to predict attachment performance purely based on the factors investigated here. A variety of factors come into play when attachment systems are used in patients, including the distribution of lever arms created by the prosthesis [48], masticatory function and parafunction, chemical and microbiological effects on materials (especially the female plastic components), and the quality of the fabrication processes.

## 4. Conclusions

This study aimed to address the ongoing issue of wear at the male–female interface in implant-supported removable prostheses by optimizing the attachment system design. The proposed flexible attachment system, featuring a short-necked superelastic Nitinol shaft, demonstrated a significant reduction in strain on the female part during prosthesis insertion. Nitinol’s superelastic properties, together with the promoted design, allow the shaft to accommodate large deformations under low plateau stress, effectively mitigating wear at the retentive interface. The optimized design achieved up to a 90% reduction in maximum strain compared to traditional solid ball anchors, indicating significant improvements in both prosthesis durability and patient comfort.

While this study presents promising results, predicting the attachment system’s clinical performance based solely on these factors remains difficult. Clinical outcomes are influenced by multiple variables, including prosthesis-induced force distribution, masticatory behavior, material interactions in the oral environment, and the quality of fabrication.

## Figures and Tables

**Table 2:** Material parameters of the Nitinol shape memory alloy were obtained in a previous study [43]. Tension–compression asymmetry refers to the ratio of the plateau stresses for forward martensitic transformation in compression to that in tension.

Param./[Unit]	$\sigma_{s}^{Cr,load}$ [MPa]	$\sigma_{f}^{Cr,load}$ [MPa]	$\sigma_{s}^{Cr,unload}$ [MPa]	$\sigma_{f}^{Cr,unload}$ [MPa]		
Values	440	450	250	240		
**Param./[unit]**	$E_{M}$ **[GPa]**	$E_{A}$ **[GPa]**	$\nu_{M}$ **[-]**	$\nu_{A}$ **[-]**	$\epsilon_{eq}^{T}$ **[-]**	**Tension–Compression Asymmetry**
Values	19	61	0.33	0.33	0.049	~1.2

**Table 3:** Material properties of polyoxymethylene (POM).

Young’s Modulus (MPa)	Poisson’s Ratio	Yield Stress (MPa)
750	0.28	60

**Table 4:** The isotropic plastic behavior of polyoxymethylene (POM) was obtained by fitting parameters into the data taken from [47].

Yield Stress (MPa)	Plastic Strain
60	0
65	0.01
70	0.04
71	0.1
72	0.3
73	0.5
74	0.8

## Data Availability

The original contributions presented in the study are included in the article, further inquiries can be directed to the corresponding author.

## Supplementary Materials

The following supporting information can be downloaded at: https://www.mdpi.com/article/10.3390/jfb15110344/s1, Video S1: Solid Titanium implant design (Figure 7); Video S2: Long Ti im-plant (Figure 8); Video S3: Embedded flexible Nitinol long shaft (Figure 9); Video S4: Embedded flexible Nitinol short shaft (Figure 10); Video S5: Short-necked embedded Nitinol shaft (Figure 11).
